# Comparison between different neoadjuvant chemotherapy regimens and local therapy alone for bladder cancer: a systematic review and network meta-analysis of oncologic outcomes

**DOI:** 10.1007/s00345-023-04478-w

**Published:** 2023-06-22

**Authors:** Abdulmajeed Aydh, Reza Sari Motlagh, Abdulaziz Alamri, Takafumi Yanagisawa, Adil Ayed, Pawel Rajwa, Ekaterina Laukhtina, Saeed M. Alasiri, Tatsushi Kawada, Hadi Mostafai, Abdulelah Ayidh, Maximilian Pallauf, Frederik König, Mohammad Abufaraj, Pierre I. Karakiewicz, Shahrokh F. Shariat

**Affiliations:** 1 Department of Urology, Comprehensive Cancer Center, Vienna General Hospital, Medical University of Vienna, Währinger Gürtel 18-20, 1090 Vienna, Austria; 2 Department of Urology, King Faisal Medical City, Abha, Saudi Arabia; 3 Men’s Health and Reproductive Health Research Center, Shahid Beheshti University of Medical Sciences, Tehran, Iran; 4 Division of Urology, Department of Surgery, King Khalid University, Abha, Saudi Arabia; 5 Department of Urology, The Jikei University School of Medicine, Tokyo, Japan; 6 Department of Family Medicine, King Khalid University, Abha, Saudi Arabia; 7 Department of Urology, Medical University of Silesia, Zabrze, Poland; 8 Institute for Urology and Reproductive Health, I. M. Sechenov First Moscow State Medical University, Moscow, Russia; 9 Department of Urology, Aseer Central Hospital, Abha, Saudi Arabia; 10 Department of Urology, Okayama University Graduate School of Medicine, Dentistry and Pharmaceutical Sciences, Okayama, Japan; 11 Research Center for Evidence-Based Medicine, Tabriz University of Medical Sciences, Tabriz, Iran; 12 Department of Radiology, King Khalid University, Abha, Saudi Arabia; 13 Department of Urology, University Hospital Salzburg, Paracelsus Medical University Salzburg, Salzburg, Austria; 14 Department of Urology, University Medical Center Hamburg-Eppendorf, Hamburg, Germany; 15 Division of Urology, Department of Special Surgery, Jordan University Hospital, The University of Jordan, Amman, Jordan; 16 The National Center for Diabetes, Endocrinology and Genetics, The University of Jordan, Amman, Jordan; 17 Cancer Prognostics and Health Outcomes Unit, University of Montreal Health Center, Montreal, Canada; 18 Department of Urology, Weill Cornell Medical College, New York, NY USA; 19 Department of Urology, University of Texas Southwestern, Dallas, TX USA; 20 Department of Urology, Second Faculty of Medicine, Charles University, Prague, Czech Republic; 21 Hourani Center for Applied Scientific Research, Al-Ahliyya Amman University, Amman, Jordan; 22 Karl Landsteiner Institute of Urology and Andrology, Vienna, Austria

**Keywords:** Bladder cancer, Chemotherapy, Cisplatin, Neoadjuvant, Radical cystectomy

## Abstract

**Purpose:**

The present systematic review and network meta-analysis (NMA) compared the current different neoadjuvant chemotherapy (NAC) regimes for bladder cancer patients to rank them.

**Methods:**

We used the Bayesian approach in NMA of six different therapy regimens cisplatin, cisplatin/doxorubicin, (gemcitabine/cisplatin) GC, cisplatin/methotrexate, methotrexate, cisplatin, and vinblastine (MCV) and (MVAC) compared to locoregional treatment.

**Results:**

Fifteen studies comprised 4276 patients who met the eligibility criteria. Six different regimes were not significantly associated with a lower likelihood of overall mortality rate compared to local treatment alone. In progression-free survival (PFS) rates, cisplatin, GC, cisplatin/methotrexate, MCV and MVAC were not significantly associated with a higher likelihood of PFS rate compared to locoregional treatment alone. In local control outcome, MCV, MVAC, GC and cisplatin/methotrexate were not significantly associated with a higher likelihood of local control rate versus locoregional treatment alone. Nevertheless, based on the analyses of the treatment ranking according to SUCRA, it was highly likely that MVAC with high certainty of results appeared as the most effective approach in terms of mortality, PFS and local control rates. GC and cisplatin/doxorubicin with low certainty of results was found to be the best second options.

**Conclusion:**

No significant differences were observed in mortality, progression-free survival and local control rates before and after adjusting the type of definitive treatment in any of the six study arms. However, MVAC was found to be the most effective regimen with high certainty, while cisplatin alone and cisplatin/methotrexate should not be recommended as a neoadjuvant chemotherapy regime.

**Supplementary Information:**

The online version contains supplementary material available at 10.1007/s00345-023-04478-w.

## Introduction

Radical cystectomy (RC) with lymphadenectomy is the gold standard treatment of muscle-invasive bladder cancer (MIBC) [1]. Nevertheless, muscle invasion remains a poor prognostic factor, probably because of occult metastasis at the time of diagnosis. Despite RC, approximately half of patients with deep MIBC develop metastatic disease within two years of diagnosis and succumb to their disease [2]. The addition of systemic treatment for locally advanced urothelial cancer has been investigated to eliminate unrecognized disseminated disease and improve outcomes. Transitional cell carcinoma of the bladder, the most common pathologic type of bladder cancer, is a chemo-sensitive disease that responds to cisplatin-based regimens, ranging from 50 to 70% in the metastatic state [3]. This makes chemotherapy an additional treatment for MIBC. Chemotherapy in MIBC can be administered preoperatively (neoadjuvant) and postoperatively (adjuvant). Neoadjuvant chemotherapy (NAC) offers advantages in tumor downstaging and eradication of micro-metastases and improving survival outcomes [4].

Several randomized clinical trials (RCTs) have shown that platinum-based combination neoadjuvant chemotherapy (NAC) can improve survival outcomes compared to locoregional treatment alone. However, these studies had considerable differences in patient numbers, patient characteristics, the type of locoregional treatment (radical cystectomy and/or radiation therapy), as well as the type and number of cycles of chemotherapy used. Therefore, pooling their results is questionable [2]. Nevertheless, current major guidelines recommend neoadjuvant regimes with a high level of evidence for eligible muscle-invasive bladder cancer (MIBC) patients before cystectomy. This recommendation is based not only on the results of three meta-analyses [5–7] but also on results from specific trials such as the updated analysis of a large phase III RCT [8–10]. In addition, there has been increasing interest in cisplatin/gemcitabine (GC) and cisplatin/carboplatin regimens due to their favorable toxicity profiles. Despite the acceptable benefits of NAC, the current literature could not recommend a ranking of different regimes in the neoadjuvant setting. The present systematic review and network meta-analysis aimed not to find the efficacy of NAC, which has already been demonstrated, but rather to indirectly compare different NAC regimens for MIBC patients and recommend the top one, while there is not which comparison.

## Method and materials

### Literature search

A protocol for this study was registered a priori on the International Prospective Register of Systematic Reviews (CRD42022343508). This systematic review and network meta-analysis (NMA) were conducted according to the Preferred Reporting Items for Systematic Reviews and Meta-analyses (PRISMA) extension statement for NMA [11].

The PubMed and Web of Science databases were searched in September 2021 to identify clinical trials reporting the oncologic outcomes of NAC in bladder cancer patients. We rerun the literature search in September 2022. Two authors independently performed a comprehensive systematic literature search. We used the following keywords in our search: “urothelial carcinoma,” “bladder cancer,” “urinary bladder urothelial carcinoma,” “neoadjuvant chemotherapy,” “neoadjuvant therapy” and “radical cystectomy.” The primary outcome of interest was the overall mortality rate (OM) and the secondary outcomes were progression-free survival (PFS) and any downstaging rates, i.e., complete response and/or partial response.

After removing duplicates, two reviewers screened, independently, the titles and abstracts. Next, any citation that either reviewer thought should be included or unclear for inclusion was identified for full-text screening. Subsequently, full texts of eligible articles were reviewed for final inclusion and data extraction. During the primary and secondary literature screenings, any discrepancies were resolved by referring to the co-authors in a Delphi consensus.

### Inclusion and exclusion criteria

We included studies that investigated non-metastatic bladder cancer patients who were treated with NAC before definitive local treatment [radical cystectomy (RC) or radiotherapy (RT)] compared with those who underwent RC or RT only to assess the differential effects on disease progression rate and mortality rate in phase III randomized studies. We excluded phase I and II clinical trials, observational studies, reviews, letters, editorials, replies from authors, case reports and articles not published in English.

### Data extraction

Two reviewers independently extracted data from each study. Extracted data included the following: Author, year, number of patients, regimens, standard arm, oncologic outcomes and follow-up. Subsequently, the number of events for oncologic outcomes was retrieved (Supplementary Table 1).

### Risk of bias assessment

The risk-of-bias (RoB) evaluation of each study was assessed according to The Cochrane Collaboration’s tool for evaluating RoB [12]. This tool assesses selection bias, performance bias, detection bias, attrition bias, reporting bias and other sources of bias. The RoB of each study was evaluated independently by two authors. Disagreements were resolved by consulting with the co-authors.

### Statistical analyses

We conducted an NMA using random and fixed effect models with a Bayesian approach to compare directly and indirectly NAC plus local therapy with local therapy alone as the standard comparator arm [13]. To assess the oncologic outcomes, arm-based analyses were performed to estimate the odds ratio (OR) and 95% credible interval (CrI) from the absolute numbers of events presented in the selected studies. In Bayesian statistics, Crl is the interval within which an unobserved value falls with a particular probability. Statistical significance was established with a two-sided *p* < 0.05 or 95% CrI that did not include a value of 1. All treatments were ranked according to the surface under the cumulative ranking curve (SUCRA) probability. Network plots were utilized to illustrate the connectivity of the treatment networks. All Bayesian statistical calculations were performed using MetaInsight software from the R package gemtc, gemtc: Network Meta-Analysis Using Bayesian Methods R package version 0.8–2. and R package BUGSNET, BUGSnet: Bayesian inference Using Gibbs Sampling to conduct NETwork meta-analysis version 1.0.3 [14].

## Results

### Search results

The literature search identified 887 unique references (Supplementary Fig. 1). Of the 20 full-text articles assessed for eligibility, five were excluded based on the selection criteria.

### Characteristics of the included studies

Fifteen studies comprising 4,276 patients met the eligibility criteria. Supplementary Table 2 summarizes the study characteristics. Three studies, published between 1991 and 1995, involved an assessment of cisplatin alone as neoadjuvant therapy compared to RC [15] or RT [16] alone. Two studies, published in 1998 and 2011, involved an evaluation of methotrexate, cisplatin and vinblastine (MCV) as neoadjuvant therapy compared with local therapy in the treatment of urothelial cancer of the bladder [17, 18]. Three studies, published in 1999, 2003 and 2014, involved an assessment of MVAC as NAC compared to RC alone [19–21]. Three studies, all published in 2002, involved an assessment of cisplatin and methotrexate as neoadjuvant therapy compared to local therapy alone [22, 23]. Nordic I study, published in 1996, assessed cisplatin and doxorubicin as NAC compared to RT and RC [24]. Finally, two studies, published in 2014, assessed GC as neoadjuvant therapy compared to RC or RT [25, 26]. Recently a head-to-head RCT has assessed MVAC versus GC in the neoadjuvant setting [27].

Unfortunately, most RCTs did not report comparative and classified toxicity side effect rates. Therefore, toxicity NMA between different NAC regimens was not feasible. The RoB for each of the trials is reported in Supplementary Fig. 2. Overall, the trials were of moderate quality, with downgrading primarily occurring for lack of blinding and bias in selecting the reported outcomes.

### Network meta-analysis

#### Comparison of the overall mortality rates

**Fig. 1:**
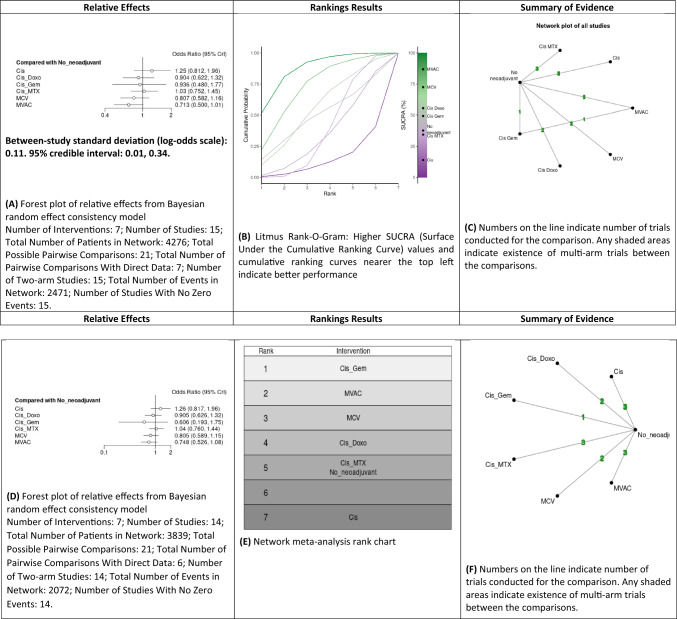
Summary of the Bayesian network meta-analysis of overall mortality rate in patients treated with neoadjuvant chemotherapy for bladder cancer (**A**–**C**) and Sensitivity analyses (**D**–**F**). Cisplatin (Cis), cisplatin/doxorubicin (Cis_Doxo), or Cisplatin and Gemcitabine (Cis_Gem), cisplatin/methotrexate (Cis_MTX), methotrexate, cisplatin and vinblastine (MCV) and Methotrexate, Vinblastine, Doxorubicin (Adriamycin), and Cisplatin MVAC

A NMA of different NAC regimens (cisplatin alone, MCV, MVAC, cisplatin/methotrexate, cisplatin/doxorubicin and GC) was conducted to assess the mortality rates (Fig. 1A–C and Supplementary Fig. 3). Compared to local treatment only, cisplatin (OR: 1.25, 95% CrI 0.81–1.96), cisplatin/doxorubicin (OR: 0.9, 95% CrI 0.62–1.32), GC (OR: 0.95, 95% CrI 0.48–1.77), cisplatin/methotrexate (OR: 1.05, 95% CrI 0.75–1.45), MCV (OR: 0.8, 95% CrI 0.58–1.16) and MVAC (OR: 0.7, 95% CrI 0.50–1.01) were not significantly associated with a lower likelihood of mortality. Based on Bayesian analysis and analysis of treatment ranking according to SUCRA, it was highly likely that MVAC appeared as the best treatment approach in terms of mortality. Cisplatin/methotrexate was superior to cisplatin only; however, both showed similar results to no NAC.

The sensitivity analysis was conducted after excluding Pfister et al. study. The overall survival (OS) result of this study was still immature due to a short follow-up [27]. A NMA of different NAC regimens (cisplatin alone, MCV, MVAC, cisplatin/methotrexate, cisplatin/doxorubicin and GC) was conducted to assess the mortality rates (Fig. 1D–F and Supplementary Fig. 3). Compared to local treatment only, cisplatin (OR: 1.25, 95% CrI 0.82–1.96), cisplatin/doxorubicin (OR: 0.9, 95% CrI 0.63–1.32), GC (OR: 0.6, 95% CrI 0.19–1.75), cisplatin/methotrexate (OR: 1.05, 95% CrI 0.76–1.44), MCV (OR: 0.8, 95% CrI 0.59–1.15) and MVAC (OR: 0.75, 95% CrI 0.53–1.08) were not significantly associated with a lower likelihood of mortality. Based on Bayesian analysis and analysis of treatment ranking according to SUCRA, it was highly likely that GC and MVAC appeared as the best treatment approach in terms of mortality. Cisplatin/methotrexate and cisplatin only; showing similar results to the main analysis.

#### Comparison of mortality rates excluding RT studies

**Fig. 2:**
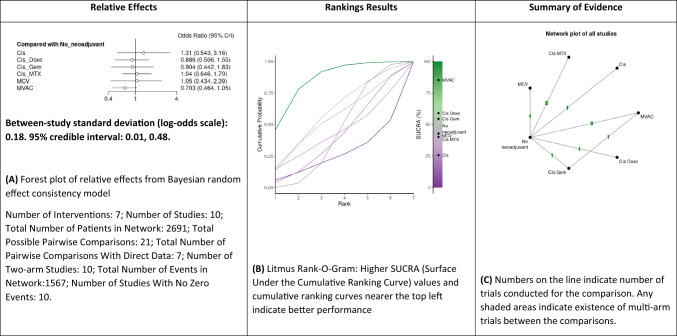
Summary of the Bayesian network meta-analysis of overall mortality rate excluding RT-only studies in patients treated with neoadjuvant chemotherapy for bladder cancer. Cisplatin (Cis), cisplatin/doxorubicin (Cis_Doxo), or Cisplatin and Gemcitabine (Cis_Gem), cisplatin/methotrexate (Cis_MTX), cisplatin, methotrexate and vinblastine (CMV) and Methotrexate, Vinblastine, Doxorubicin (Adriamycin), and Cisplatin MVAC

A NMA of different neoadjuvant therapy regimens (cisplatin alone, MCV, MVAC, cisplatin/methotrexate, cisplatin/doxorubicin and GC) was conducted to assess mortality excluding RT-only treatment studies in patients with MIBC (Fig. 2A–C and Supplementary Fig. 4). Compared to RC, with or without RT, cisplatin (OR: 1.3, 95% CrI 0.54–3.16), cisplatin/doxorubicin (OR: 0.9, 95% CrI 0.50–1.55), GC (OR: 0.9, 95% CrI 0.44–1.83), cisplatin/methotrexate (OR: 1.05, 95% CrI 0.64–1.70), CMV (OR: 1.05, 95% CrI 0.43–2.39) and MVAC (OR: 0.7, 95% CrI 0.46–1.05) were not significantly associated with a lower likelihood of mortality. Based on Bayesian analysis and analysis of treatment ranking according to SUCRA, it was highly likely that MVAC, cisplatin/doxorubicin and GC were higher than RC alone, respectively and MCV, Cisplatin/methotrexate and cisplatin were lower than to RC alone, respectively.

#### Comparison of progression-free survival rate

**Fig. 3:**
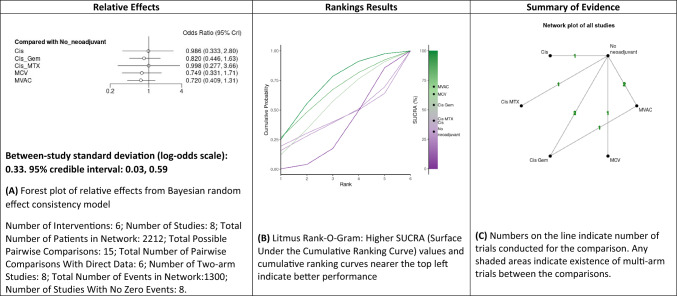
Summary of the Bayesian network meta-analysis of progression-free survival rate in patients treated with neoadjuvant chemotherapy for bladder cancer. rate (PFS). Cisplatin (Cis), cisplatin/doxorubicin (Cis_Doxo), or Cisplatin and Gemcitabine (Cis_Gem), cisplatin/methotrexate (Cis_MTX), cisplatin, methotrexate and vinblastine (CMV) and Methotrexate, Vinblastine, Doxorubicin (Adriamycin), and Cisplatin MVAC

A NMA of different NAC regimens (cisplatin alone, MCV, MVAC, cisplatin/methotrexate and GC) to assess the PFS rate was conducted (Fig. 3 and Supplementary Fig. 5). As compared with no neoadjuvant chemotherapy, cisplatin (OR: 1, 95% CrI 0.33–2.80), GC (OR: 0.8, 95% CrI 0.44–1.83), cisplatin/methotrexate (OR: 1, 95% CrI 0.27–3.66), MCV (OR: 0.75, 95% CrI 0.33–1.71) and MVAC (OR: 0.7, 95% CrI 0.4–1.31) were not significantly associated with a higher likelihood of PFS rate. Based on Bayesian analysis and analysis of the treatment ranking according to SUCRA, it was highly likely that MVAC appeared as the best treatment approach for PFS. Cisplatin/methotrexate and cisplatin solely regimens were inferior to MCV and GC.

#### Comparison of progression-free survival rate excluding RT-only studies

**Fig. 4:**
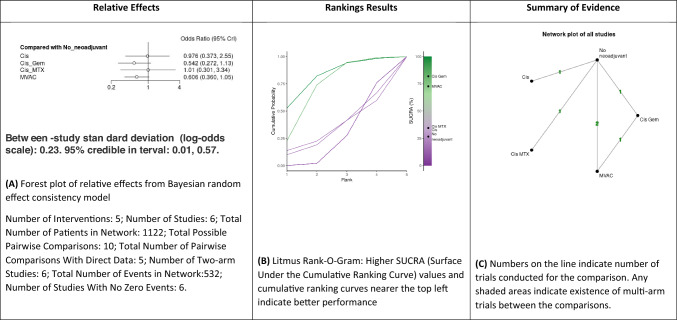
Summary of the Bayesian network meta-analysis of progression-free survival rate excluding RT-only studies in patients treated with neoadjuvant therapy for bladder cancer. Cisplatin (Cis), cisplatin/doxorubicin (Cis_Doxo), or Cisplatin and Gemcitabine (Cis_Gem), cisplatin/methotrexate (Cis_MTX), cisplatin, methotrexate and vinblastine (CMV) and Methotrexate, Vinblastine, Doxorubicin (Adriamycin), and Cisplatin MVAC

A NMA of four NAC regimens (cisplatin alone, MVAC, cisplatin/methotrexate and GC) was conducted to assess the PFS rate excluding RT-only treatment studies (Fig. 4A–C and supplementary Fig. 6). Compared to RC, cisplatin (OR: 1, 95% CrI 0.37–2.55), GC (OR: 0.55, 95% CrI 0.27–1.13), cisplatin/methotrexate (OR: 1, 95% CrI 0.30–3.34) and MVAC (OR: 0.6, 95% CrI 0.36–1.05) were not significantly associated with a higher likelihood of PFS rate. Based on Bayesian analysis and analysis of the treatment ranking according to SUCRA, it was highly likely that GC and MVAC appeared as the best treatment approach in terms of PFS rate. Cisplatin and cisplatin/methotrexate showed similar results to no NAC (i.e., RC only).

#### Comparison of local control rate

A NMA of four neoadjuvant therapy regimens (GC, cisplatin/methotrexate, MCV and MVAC) was conducted to assess any local control in patients with MIBC (supplementary Fig. 7 and 8). Compared to RC, GC (OR: 0.85, 95% CrI 0.19–5.95), cisplatin/methotrexate (OR: 2.8, 95% CrI 0.54–14.6), MCV (OR: 0.8, 95% CrI 0.14–4.15) and MVAC (OR: 0.75, 95% CrI 0.17–3.55) were not significantly associated with a higher likelihood of local control (i.e., complete response and any downstaging). Based on Bayesian analysis and analysis of the treatment ranking according to SUCRA, it was highly likely that MVAC appeared with high certainty of results as the best treatment approach in terms of local control. While MCV and GC showed to be in a lower rank than MVAC, respectively. While cisplatin/methotrexate is lower than no NAC chemotherapy.

## Discussion

We performed a systematic review and NMA of phase III RCTs to compare the mortality and oncologic/pathologic outcomes of different NAC regimens in patients with MIBC. We did not find a significant difference in terms of mortality or PFS rates among the different NAC regimes compared to local therapy only (i.e., RC and/or RT). Although this result goes against current systematic reviews and meta-analyses that found a significant survival benefit in favor of NAC [5–7], the present network meta-analysis aimed to compare different NAC regimes. In contrast to these meta-analyses, we did not combine randomized control trials with different types of definitive treatment (cystectomy and/or radiation therapy) or types of chemotherapy. Due to fewer trials in each arm of the network, an insignificant credibility interval was predictable. Nevertheless, MVAC regime was the most effective with high certainty of result according to the SUCRA ranking analysis regarding overall mortality and PFS in all main, sensitivity as well as subgroup analyses.

Regarding the second option after MVAC, there were no consistent results. MCV reached a higher rank versus GC and cisplatin/doxorubicin in the main overall mortality and PFS analyses. While in the sensitivity and subgroup analyses (i.e., excluding only RT studies) it seems that GC and cisplatin/doxorubicin could be the best second option. MCV was the popular regime in the RCTs that used RT as a definitive or additive therapy with RC. Although a safety analysis was not feasible in the present NMA, a recent head-to-head RCT showed that most of the adverse events with grade ≥ 3 toxicities were hematologic, 52% in MVAC versus 55% in GC, moreover, severe anemia, asthenia and gastrointestinal disorders with nausea and vomiting were significantly higher in MVAC compared to GC [27].

A meta-analysis has revealed an OS benefit to cisplatin-based NAC compared to local therapy alone with a 5% improvement in survival at five years and a 9% absolute improvement in 5-year disease-free survival [28]. Nevertheless, the currently available data suffer from several limitations such as the heterogeneity disease characteristics (e.g., clinical T-stages included), the type of definitive local therapy (i.e., RC and/or RT) and different combinations of cisplatin-based chemotherapy. Thus still, there is no consensus regarding the optimal NAC regime in terms of survival and oncologic outcomes. The current NMA has made an effort to diminish the heterogeneity of local therapy in the trials regarding RC as the currently recommended therapy in non-metastatic MIBC. Consequently, after excluding RT-only locoregional therapy, we found no obvious different results regarding the best-ranked NAC option, i.e., MVAC in the main NMA analysis.

Our study revealed that some regimes such as cisplatin alone and cisplatin/methotrexate should not be recommended due to their results in both relative effects and ranking analyses. The results in all main and subgroup analyses were similar and/or lower than local therapy alone. On the other hand, currently, MVAC and GC are the most utilized NAC regimes [29, 30] and we found MVAC with a high certainty of results in direct and indirect analyses is the optimal chemotherapeutic regime. Although it has been reported that MVAC compared to GC has a better PFS and OS [27, 31], OS result of the VESPER trial is still immature owing to a short follow-up lower than 5 years. Our results were inconsistent regarding overall mortality in the main and sensitivity analyses after excluding VESPER trial [27]. Altogether, with the low certainty of results and a direct analysis result between MVAC and GC, it appears that MVAC with the high level of evidence is the most favorable regime.

Few included trials reported local control outcomes such as complete response (T0) and downstaging; however, there was no significant relative effects difference in NMA of four different regimes. MVAC was still the best NAC regime in the ranking analysis to induce downstaging and/or complete response with high certainty of results. Although a cohort study reported that MVAC has a significantly better complete response rate compared to GC, the VESPER trial could not find such a significant result [27, 31]. The clinical impact of higher local control (e.g., complete response, downstaging and organ confined) and subsequent cT0 on OS are not well determined. The pathologic staging after RC is the best prognostic indicator of positive clinical and survival outcomes [32]; while there is not enough evidence that complete response after NAC, i.e., cT0 could sequence in more favorite pathologic staging, i.e., pT0 [33]. We found the consistent results between lower mortality, higher PFS rates (i.e., survival outcomes) and higher local control rate. Thus, it might be shown that a higher local control has a potential impact on the overall and oncologic survival outcomes.

While there is no consensus in terms of the best NAC regime as well as the best patient characteristics to reach higher survival outcomes, some phase II trials were conducted to assess immune checkpoint inhibitors (ICIs) or PD1/PDL1 inhibitors in a mono and combination therapy with other ICIs and/or chemotherapy [34]. The preliminary results of the phase II trials of various mono and/or combination regimes seem promising to improve survival outcomes with one-year OS and PFS between 81 to 92% and 70% to 88%, respectively. However, the side effects and survival benefits should compare with current recommended cisplatin base NAC such as MVAC and GC and/or their efficacy assessed in a phase III trial. Currently, ICIs in the neoadjuvant setting are only limited to ineligible cisplatin MIBC patients to include in the trials [35, 36].

To our knowledge, the present NMA is the first study aimed to rank different NAC regimes including only phase III RCTs. We conducted a well-designed methodological plan, consequently diminishing the heterogeneity of the literature in the neoadjuvant setting. However, still, there was some limitation to acknowledge. While the clinical impact of higher local control on survival outcomes is not well determined, few RCTs reported this outcome; therefore, our analysis and results should be considered cautiously. Additionally, the type of local therapy could influence the outcomes and RT solely is not a recommended local therapy in MIBC; therefore, we excluded the RT-only trials in the subgroup analyses to diminish the bias of design. However, still some RCTs included both RC and RT as local therapy. Although it was interesting to make a NMA comparison among different chemotherapy regimes in terms of adverse events and safety, it was not feasible due to the scarcity of data.

## Conclusions

The current analysis of phase III RCTs showed no significant differences in relative effects of six different NAC treatments before and after adjusting for the type of definitive treatment. However, in the overall and subgroup ranking analyses using the SUCRA method, MVAC demonstrated the highest certainty of results and was the best NAC treatment in terms of overall mortality, PFS and local control rates. GC and cisplatin/doxorubicin were the second-best options as NAC treatments before RC, but the certainty of their results was low. MCV was the popular NAC regimen used in RCTs that used definitive RT. Additionally, our results indicated that cisplatin alone and cisplatin/methotrexate should not be recommended as NAC treatments due to similar outcomes to local therapy alone.

## Supplementary Information

Below is the link to the electronic supplementary material.

Supplementary file1 (DOCX 45 KB)

Supplementary file2 (DOCX 818 KB)
