# Supplementary material for: Comparison between different neoadjuvant chemotherapy regimens and local therapy alone for bladder cancer: a systematic review and network meta-analysis of oncologic outcomes
Source: World J Urol. 2023 Jun 22;41(8):2185–94. doi: 10.1007/s00345-023-04478-w (PMC10415490; doi:10.1007/s00345-023-04478-w)
Supplement: Supplementary file 1 — Supplementary file1 (DOCX 45 KB) [file 345_2023_4478_MOESM1_ESM.docx]

| **Supplementary Table 1** | | | | | | |
| --- | --- | --- | --- | --- | --- | --- |
| Number of events | | | | | | |
|  | Overall mortality rate | | progression-free survival | | Downstage | |
|  | NAC | Control | NAC | Control | NAC | Control |
| Wallace et al. | 46/83 | 41/76 | N/A | N/A | N/A | N/A |
| Raghavan et al. | 25/42 | 26/54 | N/A | N/A | N/A | N/A |
| Martinez-Piñerio et al. | 43/62 | 38/60 | 41/62 | 40/60 | N/A | N/A |
| Shipley et al. | 31/61 | 31/62 | N/A | N/A | CR 24 | 28/62 |
| Bassi et al. | 53/102 | 60/104 | N/A | N/A | N/A | N/A |
| DAVECA 8901 | 6/17 | 9/16 | 10/17 | 10/16 | N/A | N/A |
| DAVECA 8902 | 49/61 | 45/59 | N/A | N/A | N/A | N/A |
| Grossman et al. | 90/153 | 100/154 | 54/153 | 77/154 | N/A | N/A |
| Nord 1 | 62/151 | 78/160 | N/A | N/A | N/A | N/A |
| Nord 2 | 73/155 | 83/154 | N/A | N/A | pT0 37/140 | 16/139 |
| BA06 30894 | 282/491 | 309/485 | 337/491 | 361/485 | N/A | N/A |
| Osman et al. | 11/30 | 15/30 | 13/30 | 17/30 | R0 26/28 | 26/30 |
| Kitamura et al. | NA/64 | NA/66 | 23/64 | 29/66 | pT0 41/59 | 55/65 |
| Khaled et al. | NA/59 | NA/55 | 40/59 | 30/55 | N/A | N/A |
| Pfister et al. | 195/218 | 204/219 | 112/218 | 106/219 | 84/199 | 71/198 |

CR = Complete response; R = Negative margin; p = pathological

| **Supplementary Table 2. Characteristics of studies reporting outcomes in patients treated with neoadjuvant chemotherapy for bladder cancer.** | | | | | | |
| --- | --- | --- | --- | --- | --- | --- |
| Study | Year | Country | Number of I versus C groups | Regimen | Standard arm | Median follow-up (Months) |
| Wallace et al. (1) | 1991 | UK. | 83 vs 76 | Cisplatin | RT | 16 |
| Raghavan et al. (1) | 1991 | Australia | 42 vs 54 | Cisplatin | RT | 16 |
| Martinez-Piñerio et al. (2) | 1995 | Spain | 62 vs 60 | Cisplatin | RC | 78.2 |
| Shipley et al. (3) | 1998 | U.S. | 61 vs 62 | MCV | RT+ Cisplatin + RC | 60 |
| Bassi et al. (4) | 1999 | Italy | 102 vs 104 | MVAC | RC | NA |
| DAVECA 8901 (5) | 2002 | Denmark | 33 vs 33 | Cisplatin + MTX | RC | NA |
| DAVECA 8902 (5) | 2002 | Denmark | 120 vs 120 | Cisplatin + MTX | RT | NA |
| Grossman et al. (6) | 2003 | U.S. | 153 vs 154 | MVAC | RC | 100.8 |
| Nord 1 (7) | 1996 | Northern Europe | 311 vs 311 | Cisplatin + doxorubicin | RT + RC | NA |
| Nord 2 (8) | 2002 | Northern Europe | 309 vs 309 | Cisplatin + MTX | RC | 63.6 |
| BA06 30894 (9) | 2011 | International | 491 vs 485 | MCV | RC or RT | 96 |
| Osman et al. (10) | 2014 | Egypt | 30 vs 30 | GC | RC | 36 |
| Kitamura et al. (11) | 2014 | Japan | 64 vs 66 | MVAC | RC | 55 |
| Khaled et al. (12) | 2014 | Egypt | 59 vs 55 | GC | RC or RT | 37.4 |
| Pfister et al. (12) | 2022 | France | 218 vs 219 | MVAC and RC and/or RT | GC and RC and/or RT | 36 |

C = Cystectomy; R/T = Radiation therapy; MTX = methotrexate; HR = hazard ratio; CMV = methotrexate, vinblastine and cisplatin;
MVAC = methotrexate, vinblastine, Adriamycin, and cisplatin; GC = Gemcitabine and cisplatin; I = Intervention; C = Control

**The Nodesplit model to assess inconsistency**

| OM   \| **comparison** \| **p.value** \| **CrI** \| \| --- \| --- \| --- \| \| d.Cis_Gem.MVAC \| 0.282150 \| NA \| \| -> direct \| NA \| -0.48 (-1.2, 0.25) \| \| -> indirect \| NA \| 0.25 (-0.89, 1.4) \| \| -> network \| NA \| -0.26 (-0.86, 0.38) \| \| d.Cis_Gem.No_neoadjuvant \| 0.258075 \| NA \| \| -> direct \| NA \| 0.57 (-0.50, 1.7) \| \| -> indirect \| NA \| -0.19 (-1.0, 0.61) \| \| -> network \| NA \| 0.093 (-0.54, 0.75) \| \| d.MVAC.No_neoadjuvant \| 0.278150 \| NA \| \| -> direct \| NA \| 0.29 (-0.070, 0.65) \| \| -> indirect \| NA \| 1.1 (-0.28, 2.4) \| \| -> network \| NA \| 0.34 (0.0063, 0.70) \| | OM without RT   \| **comparison** \| **p.value** \| **CrI** \| \| --- \| --- \| --- \| \| d.Cis_Gem.MVAC \| 0.304125 \| NA \| \| -> direct \| NA \| -0.49 (-1.3, 0.33) \| \| -> indirect \| NA \| 0.24 (-0.93, 1.5) \| \| -> network \| NA \| -0.25 (-0.91, 0.43) \| \| d.Cis_Gem.No_neoadjuvant \| 0.308050 \| NA \| \| -> direct \| NA \| 0.56 (-0.56, 1.7) \| \| -> indirect \| NA \| -0.19 (-1.1, 0.73) \| \| -> network \| NA \| 0.10 (-0.60, 0.84) \| \| d.MVAC.No_neoadjuvant \| 0.291325 \| NA \| \| -> direct \| NA \| 0.29 (-0.12, 0.71) \| \| -> indirect \| NA \| 1.1 (-0.35, 2.5) \| \| -> network \| NA \| 0.35 (-0.036, 0.78) \| |
| --- | --- | --- | --- | --- | --- | --- | --- | --- | --- | --- | --- | --- | --- | --- | --- | --- | --- | --- | --- | --- | --- | --- | --- | --- | --- | --- | --- | --- | --- | --- | --- | --- | --- | --- | --- | --- | --- | --- | --- | --- | --- | --- | --- | --- | --- | --- | --- | --- | --- | --- | --- | --- | --- | --- | --- | --- | --- | --- | --- | --- | --- | --- | --- | --- | --- | --- | --- | --- | --- | --- | --- | --- | --- | --- | --- | --- | --- | --- | --- |

| \| **comparison** \| **p.value** \| **CrI** \| \| --- \| --- \| --- \| \| d.Cis_Gem.MVAC \| 0.232450 \| NA \| \| -> direct \| NA \| 0.12 (-0.68, 0.93) \| \| -> indirect \| NA \| -0.63 (-1.6, 0.43) \| \| -> network \| NA \| -0.13 (-0.83, 0.50) \| \| d.Cis_Gem.No_neoadjuvant \| 0.229725 \| NA \| \| -> direct \| NA \| -0.12 (-0.91, 0.68) \| \| -> indirect \| NA \| 0.63 (-0.42, 1.7) \| \| -> network \| NA \| 0.19 (-0.49, 0.80) \| \| d.MVAC.No_neoadjuvant \| 0.213725 \| NA \| \| -> direct \| NA \| 0.51 (-0.14, 1.1) \| \|  \|  \|  \| \| -> indirect \| NA \| -0.25 (-1.3, 0.90) \| \| -> network \| NA \| 0.32 (-0.27, 0.91) \|   PFS | PFS without RT   \| **comparison** \| **p.value** \| **CrI** \| \| --- \| --- \| --- \| \| d.Cis_Gem.MVAC \| 0.938250 \| NA \| \| -> direct \| NA \| 0.11 (-0.65, 0.88) \| \| -> indirect \| NA \| 0.066 (-1.3, 1.4) \| \| -> network \| NA \| 0.11 (-0.51, 0.72) \| \| d.Cis_Gem.No_neoadjuvant \| 0.909325 \| NA \| \| -> direct \| NA \| 0.55 (-0.64, 1.8) \| \| -> indirect \| NA \| 0.63 (-0.36, 1.6) \| \| -> network \| NA \| 0.61 (-0.12, 1.3) \| \| d.MVAC.No_neoadjuvant \| 0.901500 \| NA \| \| -> direct \| NA \| 0.51 (-0.11, 1.1) \| \| -> indirect \| NA \| 0.42 (-1.0, 1.9) \| \| -> network \| NA \| 0.50 (-0.045, 1.0) \| |
| --- | --- | --- | --- | --- | --- | --- | --- | --- | --- | --- | --- | --- | --- | --- | --- | --- | --- | --- | --- | --- | --- | --- | --- | --- | --- | --- | --- | --- | --- | --- | --- | --- | --- | --- | --- | --- | --- | --- | --- | --- | --- | --- | --- | --- | --- | --- | --- | --- | --- | --- | --- | --- | --- | --- | --- | --- | --- | --- | --- | --- | --- | --- | --- | --- | --- | --- | --- | --- | --- | --- | --- | --- | --- | --- | --- | --- | --- | --- | --- | --- | --- | --- |

| Downstaging   \| **comparison** \| **p.value** \| **CrI** \| \| --- \| --- \| --- \| \| d.Cis_Gem.MVAC \| 0.084375 \| NA \| \| -> direct \| NA \| 0.27 (-1.1, 1.7) \| \| -> indirect \| NA \| -2.6 (-6.6, 0.41) \| \| -> network \| NA \| -0.13 (-1.7, 1.1) \| \| d.Cis_Gem.No_neoadjuvant \| 0.081400 \| NA \| \| -> direct \| NA \| -1.7 (-5.2, 0.85) \| \| -> indirect \| NA \| 1.2 (-0.94, 3.3) \| \| -> network \| NA \| 0.15 (-1.9, 1.6) \| \| d.MVAC.No_neoadjuvant \| 0.083425 \| NA \| \| -> direct \| NA \| 0.91 (-0.67, 2.5) \| \| -> indirect \| NA \| -1.9 (-5.6, 0.92) \| \| -> network \| NA \| 0.30 (-1.3, 1.7) \| |  |
| --- | --- | --- | --- | --- | --- | --- | --- | --- | --- | --- | --- | --- | --- | --- | --- | --- | --- | --- | --- | --- | --- | --- | --- | --- | --- | --- | --- | --- | --- | --- | --- | --- | --- | --- | --- | --- | --- | --- | --- | --- |

1. WALLACE DMA, RAGHAVAN D, KELLY KA, SANDEMAN TF, CONN IG, TERIANA N, et al. Neo-adjuvant (Pre-emptive) Cisplatin Therapy in Invasive Transitional Cell Carcinoma of the Bladder. 1991;67(6):608-15.

2. Martinez-Piñeiro JA, Gonzalez Martin M, Arocena F, Flores N, Roncero CR, Portillo JA, et al. Neoadjuvant cisplatin chemotherapy before radical cystectomy in invasive transitional cell carcinoma of the bladder: a prospective randomized phase III study. The Journal of urology. 1995;153(3 Pt 2):964-73.

3. Shipley WU, Winter KA, Kaufman DS, Lee WR, Heney NM, Tester WR, et al. Phase III trial of neoadjuvant chemotherapy in patients with invasive bladder cancer treated with selective bladder preservation by combined radiation therapy and chemotherapy: initial results of Radiation Therapy Oncology Group 89-03. J Clin Oncol. 1998;16(11):3576-83.

4. Bassi P, Pappagallo GL, Sperandio P, Monfardini S, Pagano F, Cosciani S, et al. Neoadjuvant M-VAC chemotherapy of invasive bladder cancer: results of a multicenter phase III trial. 1999;161(4S).

5. Sengeløv L, von der Maase H, Lundbeck F, Barlebo H, Colstrup H, Engelholm SA, et al. Neoadjuvant chemotherapy with cisplatin and methotrexate in patients with muscle-invasive bladder tumours. Acta oncologica (Stockholm, Sweden). 2002;41(5):447-56.

6. Grossman HB, Natale RB, Tangen CM, Speights VO, Vogelzang NJ, Trump DL, et al. Neoadjuvant chemotherapy plus cystectomy compared with cystectomy alone for locally advanced bladder cancer. The New England journal of medicine. 2003;349(9):859-66.

7. Rintala E, Hannisdahl E, Fosså SD, Hellsten S, Sander S. Neoadjuvant chemotherapy in bladder cancer: a randomized study. Nordic Cystectomy Trial I. Scandinavian journal of urology and nephrology. 1993;27(3):355-62.

8. Sherif A, Rintala E, Mestad O, Nilsson J, Holmberg L, Nilsson S, et al. Neoadjuvant cisplatin-methotrexate chemotherapy for invasive bladder cancer -- Nordic cystectomy trial 2. Scandinavian journal of urology and nephrology. 2002;36(6):419-25.

9. International Collaboration of T, Medical Research Council Advanced Bladder Cancer Working P, European Organisation for R, Treatment of Cancer Genito-Urinary Tract Cancer G, Australian Bladder Cancer Study G, National Cancer Institute of Canada Clinical Trials G, et al. International phase III trial assessing neoadjuvant cisplatin, methotrexate, and vinblastine chemotherapy for muscle-invasive bladder cancer: long-term results of the BA06 30894 trial. J Clin Oncol. 2011;29(16):2171-7.

10. Osman MA, Gabr AM, Elkady MS. Neoadjuvant chemotherapy versus cystectomy in management of stages II, and III urinary bladder cancer. Archivio italiano di urologia, andrologia : organo ufficiale [di] Societa italiana di ecografia urologica e nefrologica. 2014;86(4):278-83.

11. Kitamura H, Tsukamoto T, Shibata T, Masumori N, Fujimoto H, Hirao Y, et al. Randomised phase III study of neoadjuvant chemotherapy with methotrexate, doxorubicin, vinblastine and cisplatin followed by radical cystectomy compared with radical cystectomy alone for muscle-invasive bladder cancer: Japan Clinical Oncology Group Study JCOG0209. Annals of oncology : official journal of the European Society for Medical Oncology. 2014;25(6):1192-8.

12. Khaled HM, Shafik HE, Zabhloul MS, Ghoneim M, Saber RA, Manie M, et al. Gemcitabine and cisplatin as neoadjuvant chemotherapy for invasive transitional and squamous cell carcinoma of the bladder: effect on survival and bladder preservation. Clinical genitourinary cancer. 2014;12(5):e233-40.
