# Supplementary material for: Comparison between different neoadjuvant chemotherapy regimens and local therapy alone for bladder cancer: a systematic review and network meta-analysis of oncologic outcomes
Source: World J Urol. 2023 Jun 22;41(8):2185–94. doi: 10.1007/s00345-023-04478-w (PMC10415490; doi:10.1007/s00345-023-04478-w)
Supplement: Supplementary file 2 — Supplementary file2 (DOCX 818 KB) [file 345_2023_4478_MOESM2_ESM.docx]

Records identified through PUBMED and Web of Science:

Search Query:

*"((bladder OR urothelial) AND (cancer OR carcinoma) OR (bladder Neoplasms [Mesh])) AND (Neoadjuvant OR chemotherapy [Mesh]) AND external beam radiotherapy OR radiotherapy [Mesh]) AND (cystectomy [Mesh])" in PubMed and "((bladder OR urothelial) AND (cancer OR carcinoma)) AND (Neoadjuvant OR chemotherapy) AND (external beam radiotherapy OR radiotherapy) AND (radical cystectomy)" in Web of Science*

(n= 2887)

Records screened
(n =527)

Records after duplicates removed
(n = 527)

## Identification

**Records excluded after title and abstract review** (n=507)

Non-relevant according to inclusion criteria (468)

Review article (18)

Case report (21)

Abstract only (78)

Other than English language (62).

## Screening

Full-text articles assessed for eligibility
(n = 20)

**Articles excluded after evaluation**

(n = 5)

Duo to trials phase lower than Ⅲ or inappropriate control group.

## Eligibility

15 Studies included in qualitative and quantitative syntheses
(n =15)

## Included

**Figure 1: The selection process of the articles to assess survival outcomes among bladder cancer who received neoadjuvant chemotherapy compared to locoregional treatment.**

**Supplementary Figure 2: Risk of bias assessment for randomized control trials that included in the systematic review and network meta-analysis.**

**
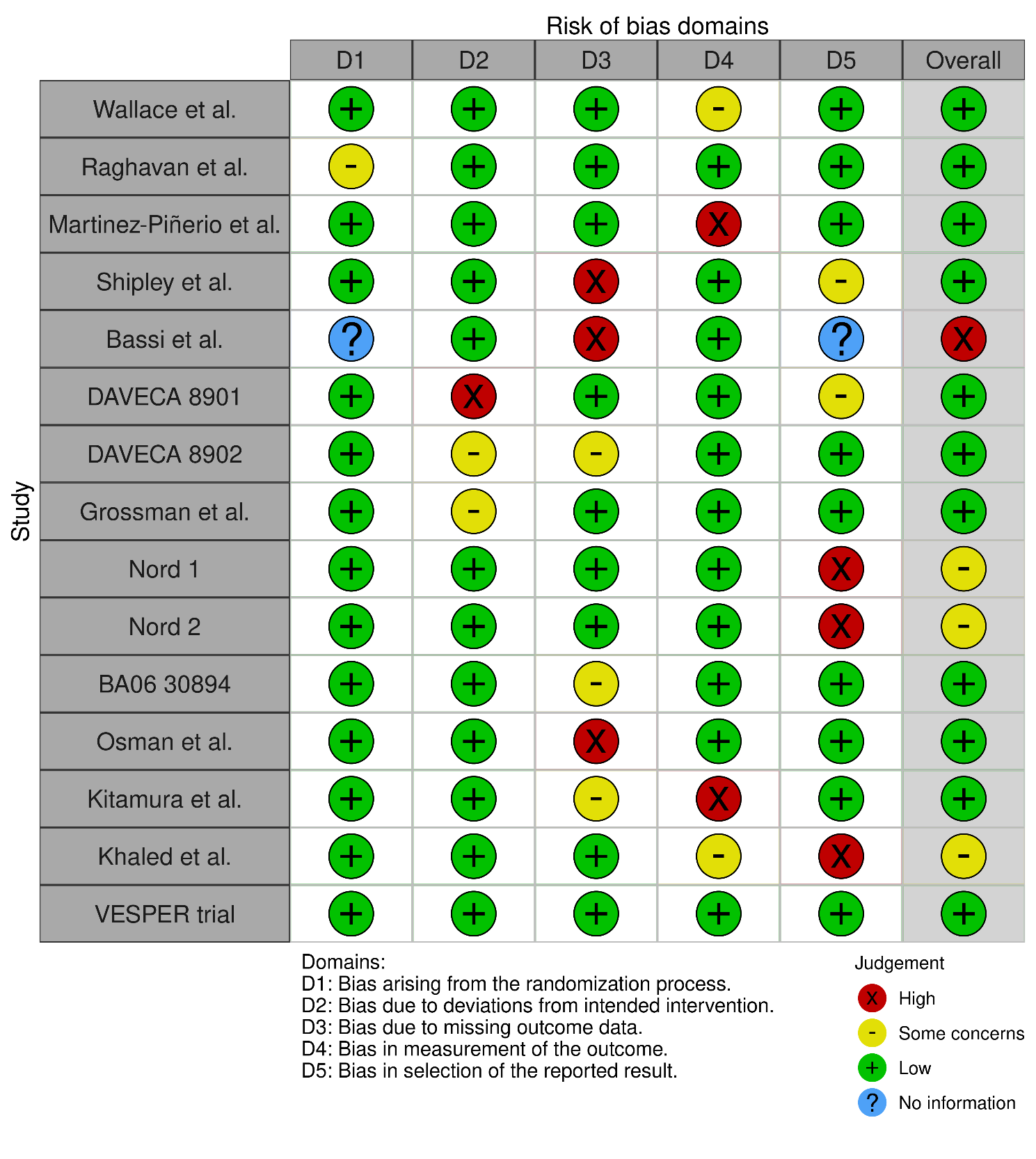
**

**Supplementary Figure 3. Main and sensitivity analyses of overall mortality rate in patients treated with neoadjuvant chemotherapy for bladder cancer.**


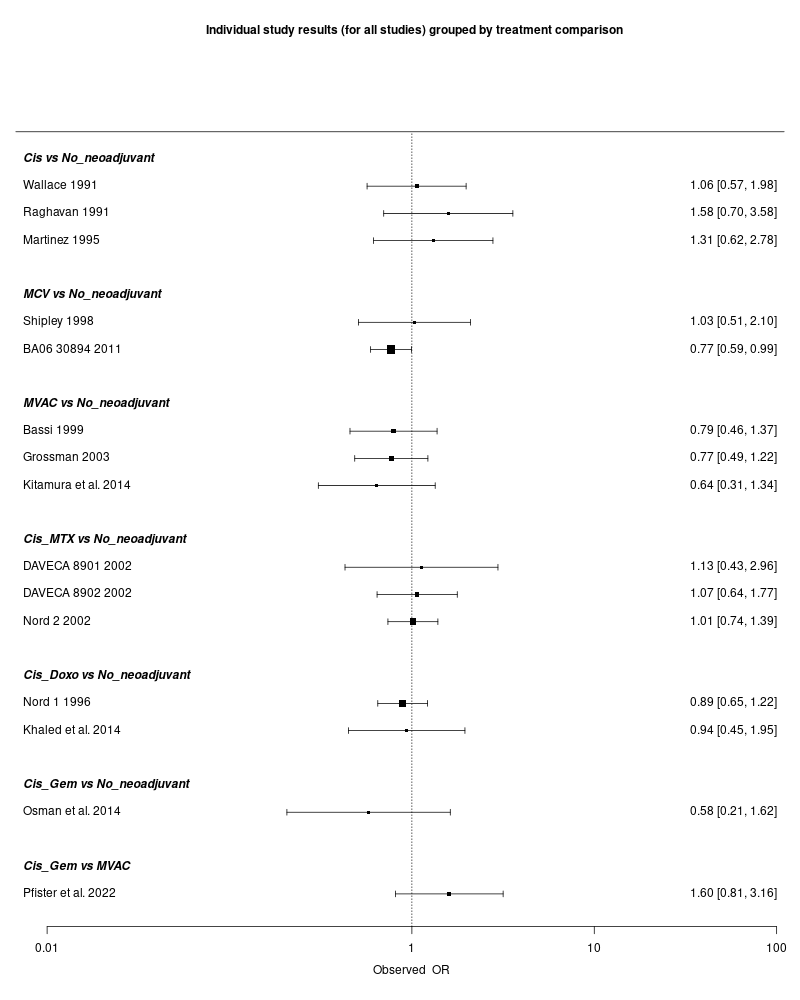


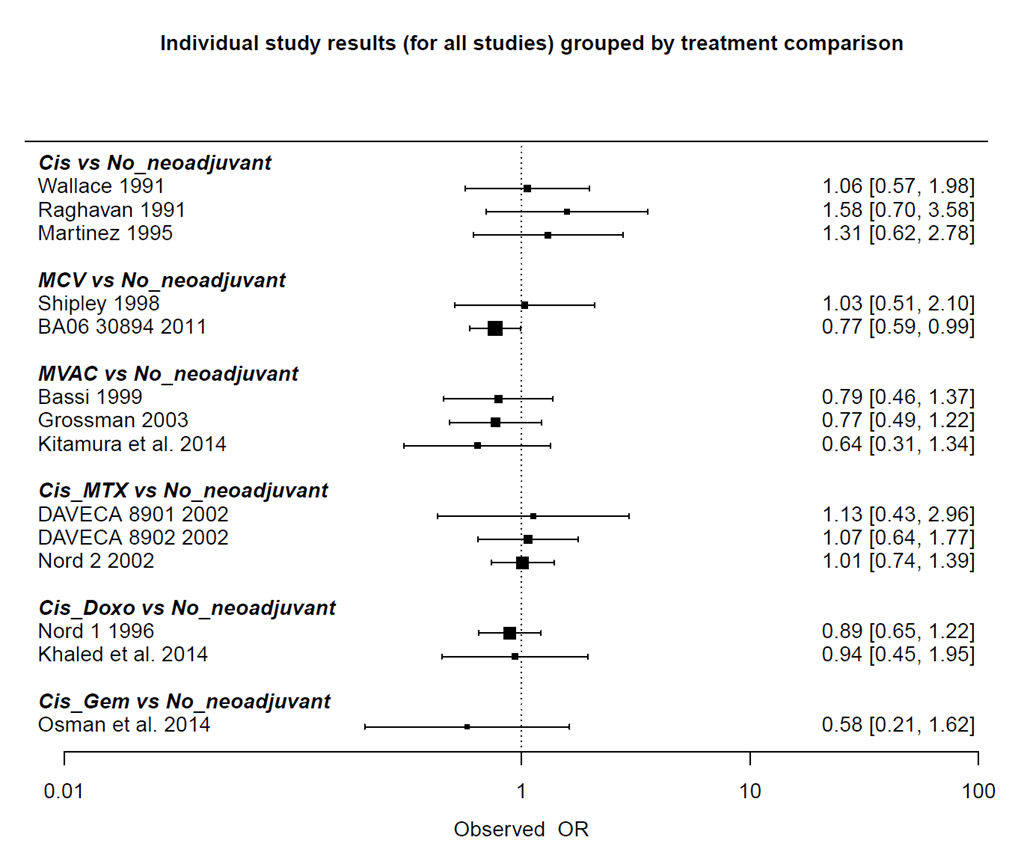


**Supplementary Figure 4. Main analysis of overall mortality rate excluding RT only studies in patients treated with neoadjuvant chemotherapy for bladder cancer.**


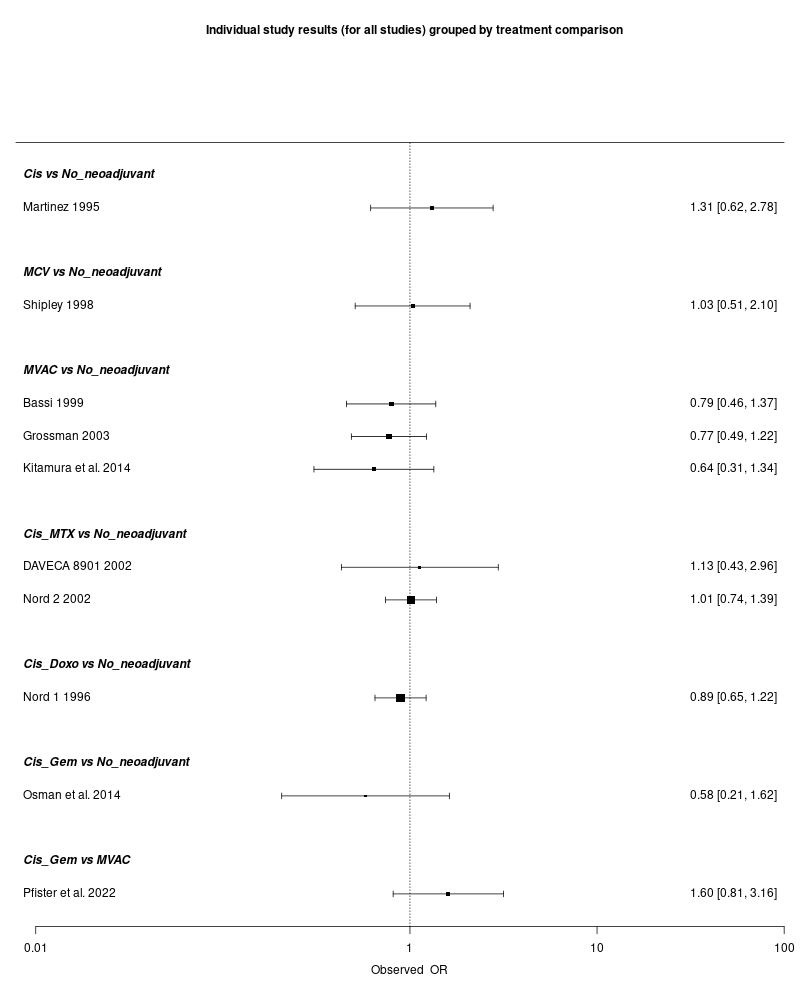


**Supplementary Figure 5. Main analysis of disease progression rate in patients treated with neoadjuvant cheomotherapy for bladder cancer.**


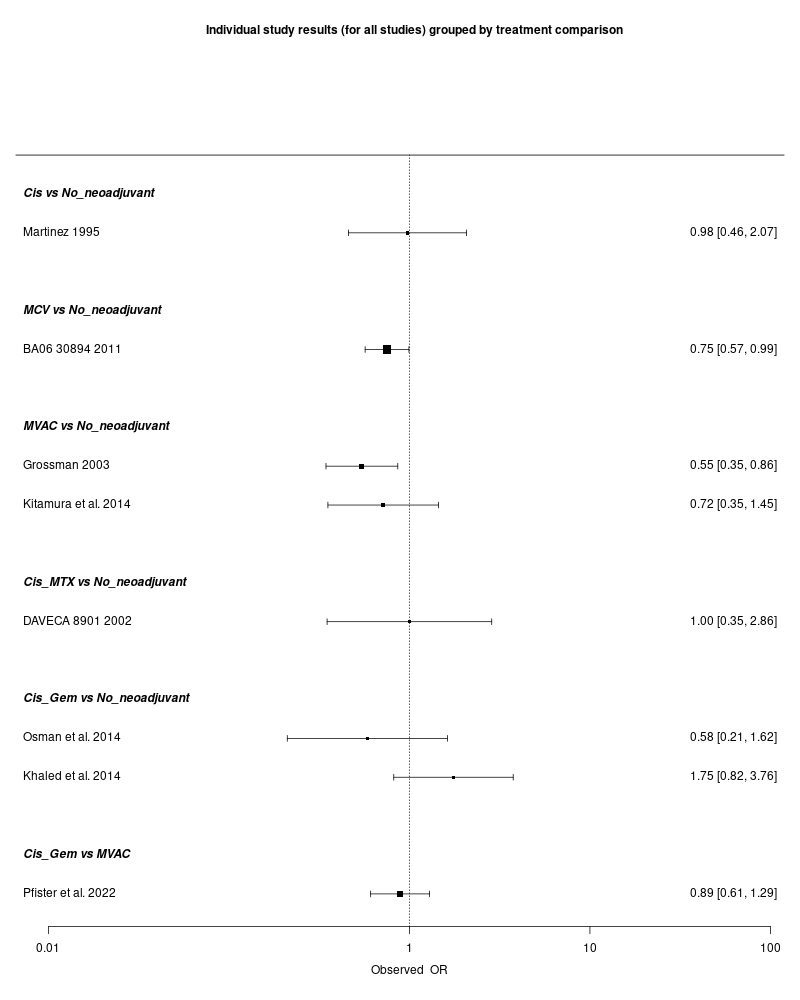


**Supplementary Figure 6. Main analysis of disease progression rate excluding RT only studies in patients treated with neoadjuvant cheomotherapy for bladder cancer.**


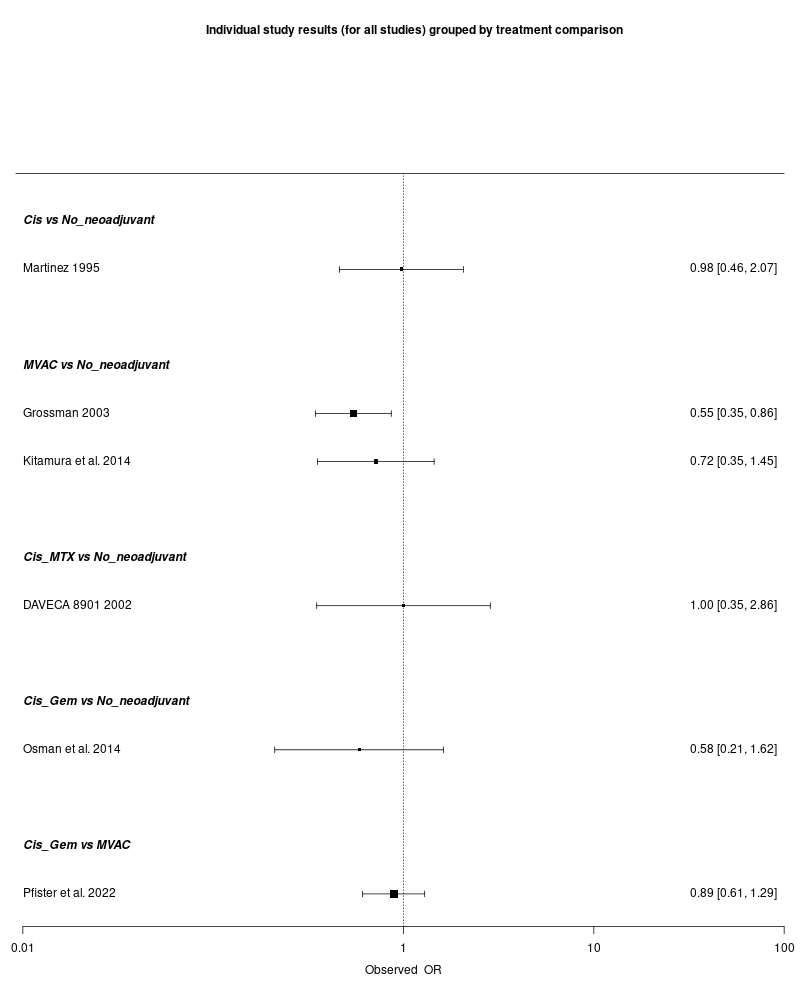
**Supplementary Figure 7. Main analysis of downstaging in patients treated with neoadjuvant cheomotherapy for bladder cancer.**


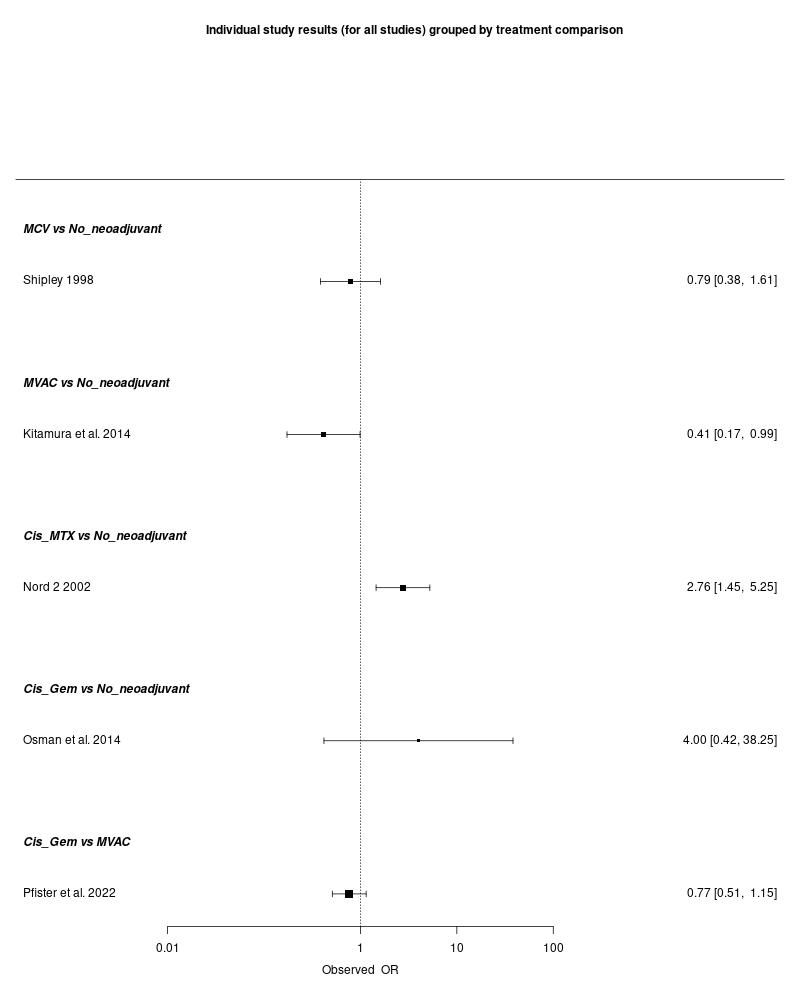


| **Relative Effects** | **Rankings Results** | **Summary of Evidence** |
| --- | --- | --- |
| 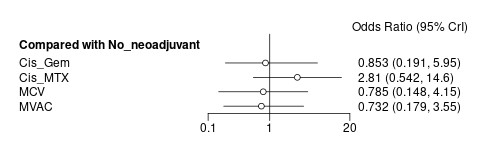  **Between-study standard deviation (log-odds scale): 0.67. 95% credible interval: 0.05, 1.08.**  **(A)** Forest plot of relative effects from Bayesian random effect consistency model  Number of Interventions: 6; Number of Studies: 8; Total Number of Patients in Network: 2212; Total Possible Pairwise Comparisons: 15; Total Number of Pairwise Comparisons With Direct Data: 6; Number of Two-arm Studies: 8; Total Number of Events in Network:1300; Number of Studies With No Zero Events: 8. | 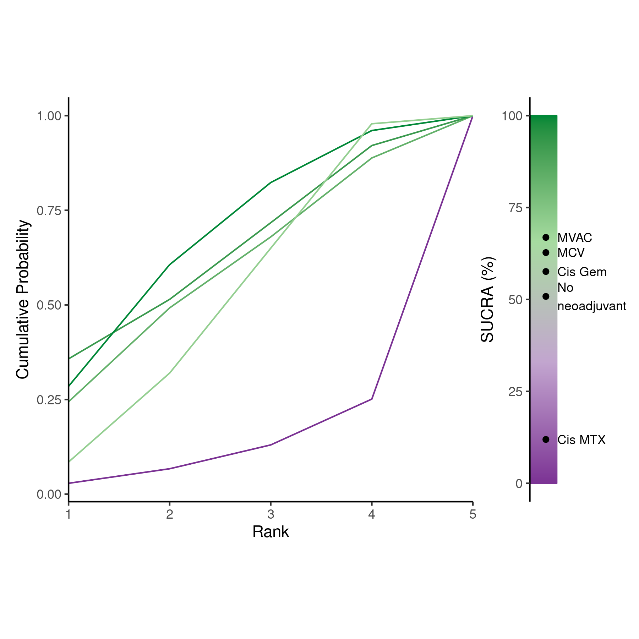  **(B)** Litmus Rank-O-Gram: Higher SUCRA (Surface Under the Cumulative Ranking Curve) values and cumulative ranking curves nearer the top left indicate better performance | 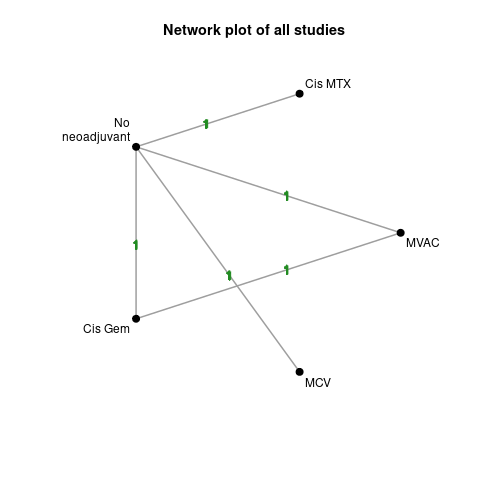  **(C)** Numbers on the line indicate number of trials conducted for the comparison. Any shaded areas indicate existence of multi-arm trials between the comparisons. |
| **Supplementary Figure 8: Summary of the Bayesian network meta-analysis of local control (i.e. complete response and/or down-staging) in patients treated with neoadjuvant chemotherapy for bladder cancer. Cisplatin (Cis), cisplatin/doxorubicin (Cis_Doxo), or Cisplatin and Gemcitabine (Cis_Gem), cisplatin/methotrexate (Cis_MTX), cisplatin, methotrexate and vinblastine (CMV) and Methotrexate, Vinblastine, Doxorubicin (Adriamycin), and Cisplatin MVAC** | | |
